# Insights into the Antimicrobial Resistance Profile of a Next Generation Probiotic *Akkermansia muciniphila* DSM 22959

**DOI:** 10.3390/ijerph19159152

**Published:** 2022-07-27

**Authors:** Daniela Machado, Joana Cristina Barbosa, Diana Almeida, José Carlos Andrade, Ana Cristina Freitas, Ana Maria Gomes

**Affiliations:** 1 CBQF—Centro de Biotecnologia e Química Fina, Laboratório Associado, Escola Superior de Biotecnologia, Universidade Católica Portuguesa, Rua Diogo Botelho 1327, 4169-005 Porto, Portugal; dmachado@ucp.pt (D.M.); jcbarbosa@ucp.pt (J.C.B.); dialmeida@ucp.pt (D.A.); afreitas@porto.ucp.pt (A.C.F.); amgomes@ucp.pt (A.M.G.); 2 TOXRUN—Toxicology Research Unit, University Institute of Health Sciences, CESPU, CRL, 4585-116 Gandra, Portugal

**Keywords:** *Akkermansia muciniphila*, antimicrobial resistance genes, antimicrobial susceptibility testing, in silico analysis, mobile genetic elements, safety profile

## Abstract

*Akkermansia muciniphila* is a Gram-negative intestinal anaerobic bacterium recently proposed as a novel probiotic candidate to be incorporated in food and pharmaceutical forms. Despite its multiple health benefits, the data addressing its antimicrobial susceptibility profile remain scarce. However, the absence of acquired resistance in probiotic strains is a compulsory criterion for its approval in the qualified presumption of safety list. This study aimed at characterizing the *A. muciniphila* DSM 22959 strain’s antimicrobial susceptibility profile using phenotypic and in silico approaches. To establish the phenotypic antimicrobial susceptibility profile of this strain, minimum inhibitory concentrations of eight antimicrobials were determined using broth microdilution and E-test methods. Additionally, the *A. muciniphila* DSM 22959 genome was screened using available databases and bioinformatics tools to identify putative antimicrobial resistance genes (ARG), virulence factors (VF), genomic islands (GI), and mobile genetic elements (MGE). The same categorization was obtained for both phenotypic methods. Resistance phenotype was observed for gentamicin, kanamycin, streptomycin, and ciprofloxacin, which was supported by the genomic context. No evidence was found of horizontal acquisition or potential transferability of the identified ARG and VF. Thus, this study provides new insights regarding the phenotypic and genotypic antimicrobial susceptibility profiles of the probiotic candidate *A. muciniphila* DSM 22959.

## 1. Introduction

Overall, probiotics are defined as “live microorganisms that, when administered in adequate amounts, confer a health benefit on the host” [1]. In the last years, the increasing knowledge regarding the pivotal role of intestinal commensal bacteria in human health has been raising the attention of the scientific community in exploiting the beneficial properties of such species in the form of probiotics [2]. In this context, *Akkermansia muciniphila* has been proposed as a next generation probiotic candidate with high potential to be incorporated in foods or pharmaceutical formulations, since it demonstrated relevant biological effects in several metabolic conditions [3,4].

Microbiologically, *A. muciniphila* is an anaerobic, Gram-negative and mucin-degrading bacterium [5], representing 1 to 3% of human faecal microbiota in healthy adults [6]. Recently, it was demonstrated that the daily oral supplementation with *A. muciniphila* improved several metabolic parameters of overweight/obese insulin-resistant human volunteers, while also being considered well-tolerated and safe [7]. 

Notwithstanding its promising results, the commercialisation of *A. muciniphila* as probiotic mandates a full assessment of safety parameters [2,8]. One important, and occasionally neglected, aspect of this safety assessment among probiotic microorganisms is the potential to carry and spread antimicrobial resistance genes (ARG) [9,10]. Indeed, antimicrobials are the main therapeutic approach for the treatment of several infectious diseases [11], while their over-prescription and misuse has contributed to the worldwide dissemination of antimicrobial resistance (AR) in pathogenic and non-pathogenic bacteria associated with humans, animals, and the environment [12,13]. Associated with the lack of discovery/development of new antimicrobial agents, AR has been considered a major global public health issue [14]. In this context, food microorganisms with intended use as probiotics are now under intense surveillance for possible involvement in the spreading of AR [15,16]. Although the existence of AR in probiotic strains could be an advantage when considering their application as adjuvants of antibiotic therapy [17], if those AR determinants have an acquired nature, the consumption of probiotics pose a potential risk of dissemination in intestinal microbiota and the environment [16,18].

In Europe, the absence of acquired genes coding for resistance against clinically relevant antimicrobials must be established for microorganisms intentionally added to food or feed in order to be declared safe for human and animal consumption and to acquire qualified presumption of safety (QPS) status from the European Food Safety Authority (EFSA) [19,20]. For this purpose, two sets of data should be provided including phenotypic susceptibility testing based on determination of a minimum inhibitory concentration (MIC) for a selected antimicrobials spectrum and the search of ARG in whole genome sequence [20]. However, few phenotypic antimicrobial susceptibility studies and in silico analyses of ARG involving *A. muciniphila* strains have been previously reported [21,22,23,24]. So, the present study aimed at characterising the antimicrobial resistance profile of next generation probiotic candidate *A. muciniphila* DSM 22959, integrating phenotypic and in silico methods.

## 2. Materials and Methods

### 2.1. Bacterial Strains and Growth Conditions

*Akkermansia muciniphila* DSM 22959 strain was obtained from Leibniz Institute DSMZ-German Collection of Microorganisms and Cell Cultures (Braunschweig, Germany). This bacterial strain was kept frozen at −80 °C in PYG broth [media composition in accordance with DSMZ recommendations [25] with minor modifications, namely: 0.05% (*m*/*v*) mucin (Sigma-Aldrich, St. Louis, MO, USA) and without resazurin] and 20% (*v*/*v*) glycerol (Fisher Chemical, Loughborough, United Kingdom). For each experiment, a cryo-stock was thawed and grown in PYG broth for 24 h at 37 °C, under anaerobic conditions (85% N_2_, 5% H_2_ and 10% CO_2_), using an anaerobic incubator (Whitley A35 HEPA anaerobic workstation, Bingley, United Kingdom). For the phenotypic antimicrobial susceptibility testing, the bacterial suspension was cultured at least once more in PYG broth.

### 2.2. Phenotypic Antimicrobial Susceptibility Testing

Minimum inhibitory concentration (MIC) of eight antimicrobials were assessed using broth microdilution and E-test methods. The broth microdilution method was performed according Clinical and Laboratory Standards Institute [26] with some minor changes. Briefly, antimicrobials recommended to be tested by the EFSA-FEEDAP guideline [20] comprised ampicillin (2–16 µg/mL), gentamicin (0.5–4 µg/mL), kanamycin (2–16 µg/mL), streptomycin (4–32 µg/mL), tetracycline (2–16 µg/mL), ciprofloxacin (0.015–0.12 µg/mL), colistin (0.5–4 µg/mL) and fosfomycin (2–16 µg/mL). These antimicrobials were obtained from Sigma-Aldrich, with the exception of ciprofloxacin and fosfomycin which were acquired from Tokyo Chemical Industry (Tokyo, Japan), and colistin that was purchased from Panreac AppliChem (Darmstadt, Germany). Antimicrobials stock solutions were made in accordance with the suppliers’ instructions. For the broth microdilution assays, an *A. muciniphila* DSM 22959 grown culture in PYG broth was used to prepare a bacterial suspension at 2 × 10^6^ CFU/mL. Then, 50 µL of two-fold serial dilutions of each antimicrobial (previously prepared in PYG broth) was placed to each well of a 96-well microplate (Sarstedt, Nümbrecht, Germany). Next, each well was inoculated with 50 µL of bacterial suspension (2 × 10^6^ CFU/mL), achieving a final bacterial concentration of 10^6^ CFU/mL. Furthermore, a negative control containing only PYG broth (100 µL/well), and a positive control containing bacterial suspension (10^6^ CFU/mL), were included. Then, the microplates were incubated anaerobically at 37 °C during 46–48 h. After incubation, the MIC, defined as the lowest antimicrobial concentration that inhibited visible bacterial growth, was evaluated. Concurrently, MIC values for these eight antimicrobials were also evaluated by E-test method following the recommendations of the manufacturer with minor modifications. In short, *A. muciniphila* DSM 22959 bacterial suspension in PYG broth with cell concentration of 1 McFarland (around 3 × 10^8^ CFU/mL) was spread uniformly onto PYG agar plates using a sterile cotton swab. After, E-test^®^ strips (Biomérieux, Marcy l’Etoile, France) containing an antimicrobial concentration gradient were placed onto the surface of inoculated PYG agar plates and incubated as described for the broth microdilution method (i.e., anaerobically at 37 °C for 46–48 h). After incubation, MIC values were read where the pointed end of the ellipse intersects the strip. Both broth microdilution and E-test assays were repeated at least three independent times with two replicates in each assay. Finally, *A. muciniphila* DSM 22959 was classified as susceptible or resistant to a specific antimicrobial in accordance of EFSA-FEEDAP microbiological cut-off values [20].

### 2.3. In Silico Analysis of Antibiotic Resistance Genes (ARG)

The presence and distribution of ARG in *A. muciniphila* species was evaluated using PATRIC—the Pathosystems Resource Integration Center [27]. A total number of 189 genomic sequences (deposited in databases until June 2021) of *A. muciniphila* strains were analysed using this software [27]. Briefly, PATRIC retrieves genome annotations and other curated data associated with each genome, including laboratory-derived antimicrobial resistance (AR) phenotypes if available, host organisms, isolation sources and geographical information. In this work, the annotated “Speciality genes” were filtered to retrieve only the “Antibiotic Resistance” related genes, whose source was PATRIC. The total number of hits was then subdivided into antibiotic related classes. 

In parallel, the genome of the strain used in this study for the phenotypic analysis, *A. muciniphila* DSM 22959 (=ATCC BAA-835, CIP 107961, JCM 33894; MUC^T^; Accession number: NZ_CP042830.1), was also analyzed using PATRIC (following the approach described above), ResFinder 3.1 [28] and CARD (version 3.1.0)—The Comprehensive Antibiotic Resistance Database [29]. Regarding the latter, a particular tool was employed, CARD’s RGI (Resistance Gene Identifier) tool (version 5.1.1) [30], which allows a broad homology-based search with defined criteria ranging from “Perfect” and “Strict” matches to “Loose” similarities.

Besides the ARG, sequences that can be indicators of possible mobilizable regions were also identified using diverse tools. Genomic islands (GI) within the *A. muciniphila* DSM 22959 genome, consisting in regions that have a putative horizontal origin, were predicted using IslandViewer4 [31]. Possible integrative and conjugative elements (ICE) and other mobile genetic elements (MGE) were detected using ICEberg 2.0 (http://db-mml.sjtu.edu.cn/ICEberg/; accessed on 21 June 2021) [32] and Mobile Element Finder [33], respectively. Prophage Hunter [34] and PHASTER [35,36] web tools were used to identify prophage-like sequences, which may indicate the integration of prophages within *A. muciniphila* DSM 22959 genome [34,35,36]. The guanine/cytosine (GC) content was determined using the genome browser Artemis (version 16.0.0) [37]. Finally, the full genome of *A. muciniphila* DSM 22959 was represented, including all of the delimited GI, integrative elements, prophage-like sequences, as well as a GC distribution graphic, using SnapGene Viewer software (from Insightful Science; available at snapgene.com accessed on 21 June 2021). 

## 3. Results and Discussion

### 3.1. Phenotypic Antimicrobial Susceptibility Profile

The MIC values of eight clinically relevant antimicrobials determined using the broth microdilution and E-test^®^ methods are presented in Table 1. Phenotypic antimicrobial susceptibility profile of *A. muciniphila* DSM 22959 was determined according to the microbiological cut-off values available in the EFSA-FEEDAP guideline (2018), which regulates the characterisation of microorganisms used as production organisms or as feed additives in the European Union. Considering the microbiological cut-off values from EFSA-FEEDAP guideline [20], the *A. muciniphila* DSM 22959 strain was categorised as susceptible to ampicillin, tetracycline, colistin, and fosfomycin. In contrast, this bacterial strain was resistant to gentamicin, kanamycin, streptomycin, and ciprofloxacin. Furthermore, the same category interpretation (susceptible or resistant) was obtained for all antimicrobials, independently of the method used (broth microdilution versus E-test). In fact, a good correlation of MIC results obtained from broth microdilution and E-test^®^ method has been previously demonstrated [38,39,40].

To the best of our knowledge, only two studies evaluated the phenotypic antimicrobial susceptibility profile of *A. muciniphila* [21,22]. In fact, Dubourg et al. performed an antibiogram for *A. muciniphila* DSM 22959 (=MUC^T^) strain using the E-test method on Wilkins–Chalgren agar plates with 5% blood. The antibiogram performed by these researchers involved different antimicrobials from those used in the present study and the interpretation was performed after five days of incubation using the EUCAST expert rules. The authors found that *A. muciniphila* DSM 22959 was susceptible to imipenem, piperacillin/tazobactam and doxycycline, being resistant to vancomycin, metronidazole and penicillin G [22]. Later, Cozzolino et al. tested the susceptibility of this strain to several antimicrobials, including chloramphenicol, ampicillin, clindamycin, tetracycline, gentamicin, streptomycin, kanamycin, and erythromycin. Using the E-test method on BHI agar plates, these researchers showed that *A. muciniphila* DSM 22959 was resistant to half of the tested antimicrobials (i.e., to chloramphenicol, clindamycin, streptomycin, and erythromycin). However, Cozzolino et al. considered the EFSA-FEEDAP microbiological cut-off values for *Lacticaseibacillus rhamnosus* (formerly *Lactobacillus rhamnosus*) [19]—Gram-positive bacteria—in the interpretation of the Gram-negative *A. muciniphila* DSM 22959 resistance phenotype, since the *L. rhamnosus* GG strain was used for comparative purposes [21]. Concerning this, the recent EFSA-FEEDAP guideline (2018) recommends Enterobacteriaceae microbiological cut-off values when evaluating Gram-negative species phenotypic antimicrobial susceptibility profile. Thus, those were the values considered in the present work. 

### 3.2. Prevalence of ARG within the Genomes of A. muciniphila Species

As genome sequencing becomes faster and cheaper, the number of genomes deposited in databases is growing exponentially every year. The analysis of genomic features is also becoming more accurate and reliable, providing a valuable complement to phenotypic studies. Concerning antimicrobial resistance traits, homology-based genome searches have been used to predict or confirm phenotypic resistance profiles [41,42]. The homology-based approach allows for the identification of virtually any known ARG as well as the identification of new variants of these genetic determinants. However, this approach retrieves only known mechanisms, while resistance caused by unknown mechanisms or resulting from gene expression modulation might remain undetected. However, genomic data can be store indefinitely and re-analysed upon phenotypic characterization of new ARG [43].

The analysis of ARG encoded in several genomes from a species allow for the establishment of a putative resistome, that is, an antimicrobial resistance profile that might be intrinsically present within that species, or even family, and is transversal to several strains [44]. In the present study, the 189 *A. muciniphila* deposited so far in PATRIC (until June 2021) were analysed to gather information regarding possible ARG. PATRIC integrates metadata publicly available from genomes deposited in GenBank [45] and RefSeq [46]. Besides the original GenBank or RefSeq annotations, each entry is also annotated using RAST (Rapid Annotations using Subsystems Technology) [47]. This way, PATRIC can be used to perform an integrated and parallel assessment of genes of interest across the different platforms [48]. PATRIC is continuously updated to facilitate the analysis of ARG within genomes, to meet the increasing demand in studying the widespread phenomena of antimicrobial resistance [49]. 

A total number of 5107 ARG were identified across all of the genomes analysed, conferring resistance to 25 classes of antimicrobial compounds (Supplementary Table S1). the effective number of ARG ranges from 18 (*A. muciniphila* strain UBA10767) to 33 (*A. muciniphila* strain AMDK-13 strain EB-AMDK-13) (Supplementary Table S2). However, on average, each genome possesses 27 annotated resistance genes, which provides an indication that most of the antimicrobial classes might be represented in all of the genomes (Supplementary Table S3). This allows the establishment of an intrinsic ARG profile that seems to be widely distributed by most, if not all, *A. muciniphila* genomes, which includes genes related with resistance against aminoglycosides (such as streptomycin), macrolides, fosfomycin, tetracyclines, and β-lactams, among others (Supplementary Table S3). Additionally, all those ARG are associated with general mechanisms of resistance, which presents as another indicator of intrinsic resistance. The ARG most frequently reported in the human gut microbiome are those related with resistance to tetracycline, β-lactams, aminoglycosides, glycopeptides, chloramphenicol, and macrolides [50]. Although all other classes are consistently represented, *A. muciniphila* seems to lack genes—or proper gene annotation—that may confer resistance to chloramphenicol and glycopeptides (Supplementary Table S3).

Production of and resistance to antibiotics are natural and ubiquitous evolutionary mechanisms that, once integrated in the microorganism’s metabolism, confer them competitive advantages in a given environment, such as the human gut [51]. It is important to note that the human microbiome is being regarded as a reservoir of well-known and new ARG, which can be genetically distant from already annotated genes [51,52]. Gut microbiota resistome is highly variable, and, just like gut microbiota itself, it depends on the hosts’ diet and environmental conditions. Consequently, a diverse ARG pattern can be found in strains isolated from different sources. On this regard, Guo and colleagues have previously analysed the whole genome of 40 *A. muciniphila* strains and revealed the horizontal acquisition of several genes, including ARG, during their co-evolution with symbiotic microbiomes in mammalian guts [24]. 

As such it is important to consider the differences between intrinsic and acquired ARG. Intrinsic resistance usually refers to ARG encoded within the chromosome which may mediate non-specific resistance mechanisms against several classes of antibiotics, including non-specific efflux pumps and inactivating enzymes (Supplementary Table S4) [53]. Acquired ARG are often obtained via horizontal gene transfer (HGT) mechanisms through mobile elements, and mediate specific resistance mechanisms, targeting a single antibiotic or antibiotic class; these include enzymes that modify the antibiotic or the antibiotic target [53]. Most ARG identified within *A. muciniphila* genomes are associated with general targets within the species or unspecific mechanisms of resistance (Table 2). Thus, they seem to consist mainly of intrinsic mechanisms of resistance, associated with, and widely distributed within, the species, rather than resistance genes acquired by HGT. 

Although in silico data provide hints regarding the establishment of a general resistome, phenotypic analysis is always required to confirm whether the predicted/annotated ARG are fully functional. To the best of our knowledge, phenotypic data regarding this species is only available for the type-strain *A. muciniphila* ATCC BAA-835 (=DSM 22959), as previously discussed [21,22].

### 3.3. ARG and Putative Mobile Elements within A. muciniphila DSM 22959 Genome

Guidelines from regulatory entities, such as EFSA-FEEDAP, encourage the use of in silico analysis to characterize possible resistance profiles of new candidate strains that are to be applied for human consumption [20]. Thus, genomic approaches are considered nowadays as major tools for antibiotic resistance surveillance, by allowing the prediction of the potential resistance profile of a particular strain of interest. 

Within this scope, *A. muciniphila* DSM 22959 genome was analysed in more detail using PATRIC [49]. A total number of 26 ARG were obtained, which include genes that might mediate resistance against β-lactams, aminoglycosides, macrolides, fluoroquinolones, and tetracyclines among others (Supplementary Table S5). Thus, there is genomic evidence supporting the phenotypic observation of resistance to gentamicin, kanamycin, streptomycin (aminoglycosides), and ciprofloxacin (fluoroquinolones) (Table 1). Interestingly, the detected ARG also support the phenotypic evidence reported by Cozzolino and co-workers, as genes encoding resistance to erythromycin (macrolide) and chloramphenicol could also be identified [21]. Still regarding the resistance to fluoroquinolones, one “Strict” hit was also obtained using CARD. This database includes molecular sequence reference data, which is continuously curated, and is used for prediction of AR genotype from genomic data. This database includes intrinsic resistance, dedicated resistance genes, and acquisition of resistance via mutation of antimicrobial targets and associated elements [30]. The RGI software predicts ARG encoded within genomes, including non-annotated sequences, using CARD-deposited ARG data as query sequences [29]. Specifically, the RGI software evaluates genomic sequences considering three paradigms: “Perfect”, “Strict”, and “Loose”. The “Perfect” algorithm detects ARG with an exact match (100%) to a reference sequence and it is typically applied for clinical surveillance. The “Strict” algorithm accommodates rather flexible sequences with some level of variation comparing to the CARD reference sequence. If a sequence falls within the curated BLAST cut-off, this threshold assures some degree of functionality of the detected ARG variant. This algorithm is useful to detect antibiotic targets altered via mutation or previously unknown variants of ARG. The “Loose” algorithm, also known as “Discovery”, works outside the cut-off of the detection model, allowing for the detection of new, emergent threats and more distant homologs of ARG, as well as homologous partial sequences that may not have a role in AR. When combined with phenotypic screening, this algorithm allows for the discovery of novel ARG [29,30]. Besides the identified “Strict” match, other 147 “Loose” hits were retrieved by RGI software upon homology-based search within *A. muciniphila* DSM 22959 genome (Supplementary Table S6). The absence of “Perfect” hits is not surprising, considering the novelty of the antibiotic resistance profiling in this species, which is increasing in recent years. The “Loose” matches comprise 19 (out of 26) hits that fit the ones previously identified with PATRIC and other new hits, corresponding to several classes of antimicrobials (Supplementary Table S5, highlighted), which might represent new variants of previously known resistance genes.

Although *A. muciniphila* DSM 22959 genome encodes resistance genes related with β-lactams, tetracyclines and fosfomycin, as shown both by PATRIC and CARD, no phenotypic evidence of resistance was observed for any of those antimicrobials (Table 1; Supplementary Tables S5 and S6). It is important to stress that the presence of a specific ARG encoded within bacterial strain genome does not necessarily translate into a resistant phenotype, for various reasons: (i) it might not be expressed in the growth conditions; (ii) the gene itself can be truncated; and (iii) the product of a specific gene might only confer a low level of resistance, thus, not reaching the cut-off values defined by EFSA-FEEDAP to classify it as a resistant strain [20]. In addition, it is important to consider that the principle underlying ARG search and annotation relies on homology-based searches; therefore, it might not be possible to guarantee that the product resulting from the detected ARG sequence retains the function of the original query sequence [42]. Previous studies have also focused the characterization of the genomic profile in terms of putative ARG. For instance, Van Passel and colleagues detected the presence of two genes encoding for β-lactamases [23]. Although both strains (BAA-835 and DSM 22959) are genetically identical, they were independently sequenced and annotated. Only one of those three genes identified in BAA-835 genome, corresponding to a β-lactamase, was annotated during the assembly of DSM 22959 genome.

Resistance genes related with colistin (or polymyxins group, in general) could not be identified with any of the tools employed. It is worth mentioning that the mechanism of action of colistin involves the solubilization of the bacterial cell membrane, through its binding to the lipid A group of lipopolysaccharides (LPS) present in the outer membrane of Gram-negative bacteria. Thus, *A. muciniphila*, as other Gram-negative bacteria, is in principle, intrinsically susceptible to colistin [54]. However, mechanisms conferring resistance against colistin were reported and usually rely on lipid A alterations or absence, either due to (i) point mutations, which might not be evident in homology searches; (ii) the presence of specific resistance genes, usually acquired through horizontal gene transfer; or (iii) deregulation of genes involved on lipid A/LPS biosynthesis [55].

Although the presence of ARG must be regarded cautiously considering the worldwide spread antibiotic resistance challenge, the real concern lies in their transmission across bacterial species [56,57]. Indeed, EFSA-FEEDAP guidelines for the evaluation of a novel probiotic strain consider acquired resistance a major safety concern that must be fully addressed in order to obtain authorization for human consumption [20]. Horizontal gene transfer between commensal and opportunistic bacteria in human gut is a well-documented phenomenon. In fact, the gut environment provides favourable conditions to conjugation events, due to the high bacterial cell density. In this context, ResFinder was used to detect putatively acquired ARG, allowing their specific detection within genomes, using a defined threshold of 98.00% identity. Thus, it detects whole resistance genes, although not providing information regarding their functional integrity and expression [58]. ResFinder did not retrieve any hit, which seems to be a safe indication of the lack of acquired resistance mechanisms.

**Figure 1:**
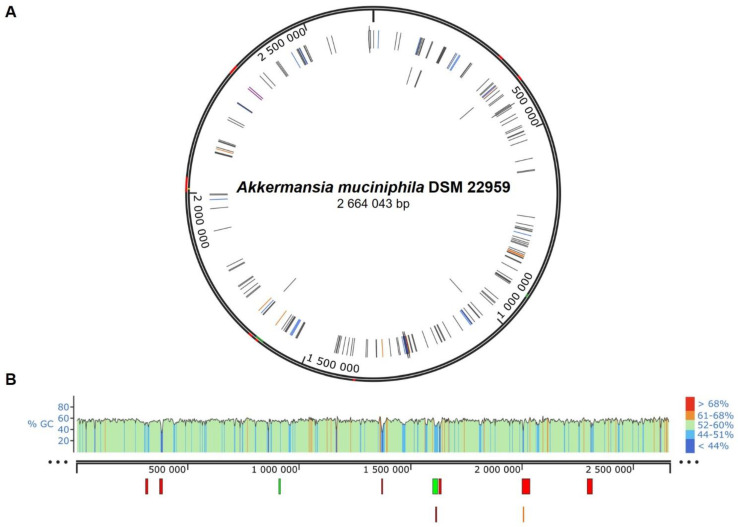
*Akkermansia muciniphila* DSM 22959 genome map. (**A**) represents the whole chromosome, with MGE (in orange), GI (in red) and Prophage-like region (in green), highlighted in the external circles; possible ARG, identified by CARD are represented inside the chromosome circle: in blue, ARG confirmed with PATRIC and CARD; in grey, ARG identified only by CARD; in orange, ARG identified only by PATRIC and in purple ARG encoded within a GI. (**B**) GI (red bars), MGE (orange bar) and prophage-like region (green bar) representation and GC content across the chromosome, with colour coded scheme, to ease the correlation. Images produced in SnapGene Viewer.

To assess the presence of regions that were possibly acquired by HGT in bacterial genomes, the presence of putative GI was investigated using IslandViewer [31]. Genomic islands are defined as clusters of consecutive genes, differing between closely related strains, that can be mobile, non-mobile or might have lost their mobility during evolution. Mobile regions might include several types of mobile genetic elements such as ICE, among others [59] and usually include mobility genes and insertion sequences (IS) that promote GI integration in the core chromosome [31,60,61]. This integration is often favoured when the sequence provides a selective advantage in terms of bacterial fitness or environmental adaptation. Genomic islands are usually characterized by a shift on the GC content in comparison with the average GC content of the chromosome where they are inserted. IslandViewer 4.0 webserver allows the prediction of GI based on several parameters that include nucleotide bias, the presence of genes associated with mobility, and comparative genomics [31]. Regarding *A. muciniphila* DSM 22959, 9 very short GI were predicted with IslandViewer, some of them overlapping each other (Figure 1A; Supplementary Table S7). Interestingly, the CG content of these regions is not substantially distinct from the average GC content of the chromosome (average GC% = 55.76; Supplementary Table S7), although some of the regions can be clearly differentiated (Figure 1B). Only GI number 9 (Supplementary Table S7) reveals the presence of 2 putative ARG, expectedly involved in general mechanisms of resistance: one identified with PATRIC, which may confer resistance against fosfomycin (Supplementary Table S5), and another CARD hit, related with resistance against peptide antibiotics (Supplementary Table S6), a class of antimicrobials that includes, for instance, daptomycin. Comparing with the phenotypic results, it is possible to conclude that the putatively acquired ARG related with fosfomycin resistance is partially or completely unfunctional, as *A. muciniphila* DSM 22959 was categorised as susceptible to fosfomycin (Table 1). 

As mentioned, the gut environment provides favourable conditions to conjugation between bacteria, promoting the spreading of plasmids and integrative and conjugative elements (ICE), which might carry ARG [56]. To date, no plasmids were reported for this strain, thus, excluding that vector as a source if mobilizable ARG. To understand the ARG transferability potential in *A. muciniphila* DSM 22959, putative ICE regions within its genome were predict using ICEfinder tool from IceBerg 2.0 software. These regions are characterized by the presence of recombination and conjugation modules, that are able to mobilize other genetic elements encoded in the vicinity, including chromosome-borne integrative and mobilizable elements (IME) [32]. No ICE regions were identified using ICEFinder. To discard any possible anomaly of the prediction software in retrieving the ICE regions, an additional tool was employed: MobileElementFinder [33]. This webserver allows the detection of integrating-MGE, through a sequence similarity-based search, using a large database of well-known MGE sequences from several different species as query sequences. The webserver excludes partial MGE, as it would not be possible to ensure the presence of the full MGE. MobileElementFinder can be used to detect several types of mobilizable elements, including the abovementioned large MGE, such as ICE and IME, as well as insertion sequences, unit and composite transposons, and miniature inverted repeats. Additionally, MobileElementFinder annotates the relationship between the detected MGE and putative ARG, virulence genes and plasmids, providing an integrated overview of the so-called mobilome of a bacterial strain [33]. This webserver detected one single MGE within the genome of *A. muciniphila* DSM 22959, corresponding to an insertion sequence (Figure 1A; Supplementary Table S8). This MGE is encoded within one of the GI detected with IslandViewer. However, no ARG are present within this region, which means that this mobilizable region does not pose any risk of transferring ARG to other bacteria (Figure 1A; Supplementary Table S7).

Bacteriophages (also known as phages) also play a major part in the mediation of HGT since they are ubiquitous in the microbial communities [62,63]. Bacteriophages are natural carriers of DNA sequences, and their life cycle is prone to carry bacterial DNA from one bacterial cell to another, regardless of the species. Briefly, the prophage is the latent form of a bacteriophage, meaning that the viral genes are assimilated in the bacterial cell, without causing its disruption, integrated either in the chromosome or in and extrachromosomal plasmid [64]. If the prophage genes are intact, it retains its lytic capabilities, that can be induced by several factors. During the lytic cycle, the new viral particles can include not only the prophage genes but also genes from the host that can then be transferred to another bacterial genome in another cycle of infection [62,63,65,66]. Bacteriophages actively contribute to the plasticity of bacterial genomes through the selection and horizontal transference of genetic information that might confer some evolutionary advantage, including virulence factors or ARG [65]. Although PATRIC did not retrieve the existence of annotated virulence factors within *A. muciniphila* DSM 22959 genome, the genome was analysed with specifically dedicated web tools, to rule out any possible risk of HGT via prophage excision. The presence of putative prophage regions was assessed using PHASTER [35] and Prophage Hunter [34]. PHASTER is an integrated web tool which allows the search and annotation of putative clusters of prophage genes, while evaluating the completeness of these prophages [35]. Only one putative prophage cluster was identified, that was classified as incomplete (Figure 1A; Supplementary Table S9). This region was also identified by Prophage Hunter (Candidate 22, Supplementary Table S10), being classified as “ambiguous”. Prophage Hunter, a machine learning platform that identifies sequences putatively encoding prophages, categorises the identified prophages as either: (*i*) “active”, if they exhibit the complete genomic sequence and thus can be induced to produce phage particles”; (*ii*) “ambiguous”, if their sequences are somehow incomplete, including mutated or truncated genes; or (*iii*) “inactive”, when the sequences present some degree of similarity but are very unlikely to yield functional prophage particles [34,66]. Prophage Hunter identified a total number of sixty-one prophage candidates, from which, only one was determined to be active that does not correspond to the one identified with PHASTER (Supplementary Table S10) but includes a region that was previously identified as a putative GI (GI number 4; Supplementary Tables S7 and S10). This indicates that this region might have been acquired through prophage integration within *A. muciniphila* DSM 22595 chromosome. Although the web tools classify the prophages as “active” or “intact” (in Prophage Hunter and PHASTER, respectively) the detected sequences might not be able to produce functional phage particles, since homology search does not ensure functionality or they might lack the proper induction factor [66]. A deeper analysis revealed the absence of putative ARG embedded in the most probable prophage regions according to both web tools, thus reducing the possibility of antimicrobial resistance spreading to other species within the environment. 

Altogether, it seems reasonable to presume that *A. muciniphila* DSM 22959 does not pose a risk of ARG horizontal transfer, as no significant mobile genetic elements were detected within its genome. In addition, *A. muciniphila* DSM 22959 reveals a very low tendency to incorporate exogenous DNA within its chromosome, since a very low number of genomic islands and prophages could be identified. More importantly, the putatively acquired or mobilizable elements do not include ARG, which reduces the risk of ARG transferability.

The transferability of chromosomal regions containing ARG depends on several factors which include, naturally, the presence of specific MGE, but also physical conditions, such as high bacterial cell density or selective pressure due to the presence of a specific antibiotic. Indeed, regarding the high fitness cost that comes with the acquisition and, specially, the fixation of ARG, these processes are very unlikely to occur without the selective pressure of an antibiotic, as no benefits will be added to compensate for the metabolic costs [67]. Thus, although the human gut may provide enough proximity between commensal/probiotic and pathogenic strains, with putative transferable ARG, horizontal transference of such genes highly depends on the antibiotic pressure selectivity, which reinforces the need of well-defined antibiotic administration practices.

## 4. Conclusions

This study presents novel data regarding the establishment of phenotypic and genotypic AR profiles of the probiotic candidate *A. muciniphila* DSM 22959. This information is of upmost importance as it is required for the acceptance of a bacterial strain to be used as food/feed additive. In this way, a phenotypic AR profile, supported by the genomic context, was established. Additionally, other ARG were detected that did not translate into a resistance phenotype. Most of the detected ARG are related to general resistance mechanisms and are not included within GI or MGE that allied with the absence of plasmids, reduce the AR transferability risk. Only two putative ARG seem to be acquired through HGT—related to fosfomycin and peptide antibiotics resistance—since they are encoded within GI. However, these regions are not likely to be mobilizable, as they are not embedded within MGE or prophage regions. Moreover, regarding the fosfomycin-related ARG seems to be unfunctional, given that no phenotypic observation of resistance is reported. The absence of acquired and putatively mobilizable ARG is a key criterium in the checklist to attribute the QPS status to a bacterial strain. Thus, future studies should focus on clarifying the transferability risks and further characterizing the antimicrobial susceptibility profile of *A. muciniphila* DSM 22959, namely by including other clinically relevant antimicrobials for humans, referring to other important guidelines such as CLSI and EUCAST. In general, the development of standardize protocols for phenotypic AR profiling and well-established pipelines for genotypic analysis, would provide an unvaluable contribution to enable comparative studies among researchers in food microbiology. In addition, it would allow a deeper safety assessment of next-generation probiotics prior to its use in food/feed products, as for the case of the candidate *A. muciniphila* DSM 22959.

## Figures and Tables

**Table 1:** Phenotypic antimicrobial susceptibility profile of *Akkermansia muciniphila* DSM 22959.

Antimicrobial	MIC (µg/mL) in Broth Microdilution	MIC (µg/mL) in E-Test^®^	EFSA-FEEDAP Cut-Off Values (µg/mL)
Ampicillin	≤2	0.25–0.75	8
Gentamicin	>**4**	**16**–**32**	2
Kanamycin	>**16**	≥**256**	8
Streptomycin	>**32**	**192**–**256**	16
Tetracycline	≤2	0.125–0.25	8
Ciprofloxacin	>**0.12**	≥**32**	0.06
Colistin	≤0.5	<0.016	2
Fosfomycin	4–8	0.5–1	8

Note: Resistances are indicated in **bold** i.e., MIC exceeding EFSA-FEEDAP cut-off values.

**Table 2:** Putative ARG detected within 189 *A. muciniphila* genomes, divided by their mode of action and antibiotic classes. In **bold** are represented mechanisms that are specific for a certain antibiotic.

Mode of Action	CARD Definition	# Hits	Antibiotics	# Hits
Antibiotic target in susceptible species	Antibiotic-sensitive wild-type bacterial components; might suffer mutations that render them not susceptible	4073	Bicyclomycin	387
			D-cycloserine, Cycloserine	569
			Brodimoprim, Iclaprim, Tetroxoprim, Trimethoprim	189
			Enacyloxin IIa, Kirromycin, Pulvomycin	193
			Ciprofloxacin, Clofazimine, Gatifloxacin, Levofloxacin, Moxifloxacin, Nalidixic acid, Ofloxacin, Sparfloxacin, Trovafloxacin	193
			Ciprofloxacin, Clofazimine, Clorobiocin, Coumermycin, Coumermycin A1, Gatifloxacin, Levofloxacin, Moxifloxacin, Nalidixic acid, Novobiocin, Ofloxacin, Sparfloxacin, Trovafloxacin	193
			Fosfomycin	197
			Fosmidomycin	190
			Fusidic acid	250
			Ethionamide, Isoniazid, Triclosan	189
			Isoniazid, Triclosan	379
			Mupirocin (Pseudomonic acid)	194
			Daptomycin	189
			Daptomycin, Rifabutin, Rifampin, Rifamycin	195
			Dapsone, Mafenide, Sulfacetamide, Sulfadiazine, Sulfadimidine, Sulfadoxine, Sulfamethizole, Sulfamethoxazole, Sulfasalazine, Sulfasoxazole	1190
			Tetracycline, Tigecycline	188
			Aminoglycosides (Streptomycin)	188
Protein altering cell wall charge conferring antibiotic resistance	Cell wall alteration	188	Peptide antibiotics (Daptomycin)	188
Gene conferring resistance via absence	Deletion of gene or gene product results in resistance. For example, deletion of a porin gene blocks drug from entering the cell.	2	Aminoglycosides (Streptomycin)	2
Antibiotic inactivation enzyme	Enzyme that catalyses the inactivation of an antibiotic resulting in resistance. Inactivation includes chemical modification, destruction, etc.	211	Beta-lactam antibiotics	187
			Lincosamides (Clindamycin, Lincomycin)	20
			Aminoglycosides (Streptomycin)	4
Efflux pump conferring antibiotic resistance	Subunits of efflux proteins that pump antibiotic out of a cell to confer resistance.	568	Acriflavin, Amikacin, Arbekacin, Cefepime, Chloramphenicol, Ciprofloxacin, Erythromycin, Gentamicin C, Meropenem, Norfloxacin, Ofloxacin, Tetracycline, Tobramycin	373
			Macrolides (Erythromycin)	194
			Tetracyclines	1
**Antibiotic target protection protein**	**These proteins confer antibiotic resistance by bind the antibiotic target to prevent antibiotic binding.**	5	Tetracyclines	5
**Antibiotic target modifying enzyme**	**Enzymes that confer resistance by modifying (mutational alteration or enzymatic modification) antibiotic targets.**	59	Azithromycin, Clarithromycin, Clindamycin, Dalfopristin, Dirithromycin, Erythromycin, Griseoviridin, Lincomycin, Madumycin II, Oleandomycin, Ostreogrycin B3, Patricin A, Patricin B, Pristinamycin IA, Pristinamycin IB, Pristinamycin IIA, Quinupristin, Roxithromycin, Spiramycin, Telithromycin, Tylosin, Vernamycin B-gamma, Vernamycin C, Virginiamycin S2	59
Regulator modulating expression of antibiotic resistance genes *	**Mechanism activated by the presence of a specific antibiotic.**	1	Multiple antibiotic resistance *	1

* Although this type of ARG is considered a specific mechanism to confer resistance against a certain class of antibiotics, the algorithm could not assign a class of antibiotics to this particular ARG.

## Data Availability

All of the data presented in this study are available within the manuscript and respective supplementary material.

## Supplementary Materials

The following supporting information can be downloaded at: https://www.mdpi.com/article/10.3390/ijerph19159152/s1, Supplementary File A and Supplementary File B, containing data regarding the in silico analysis of the *A. muciniphila* genomes and the *A. muciniphila* DSM 22959 genome, respectively.
